# COVID-19 induces greater difficulty in blood pressure control due to increased arterial stiffness

**DOI:** 10.1007/s11739-025-04138-4

**Published:** 2025-11-05

**Authors:** Marialuisa S. Marozzi, Ilaria Fucile, Immacolata Panettieri, Mauro Pagani, Rosa Curcio, Francesco Corvasce, Giuseppe S. Falcone, Costantino Mancusi, Raffaele Izzo, Angelo Vacca, Sebastiano Cicco

**Affiliations:** 1 Unit of Internal Medicine “Guido Baccelli”, Department of Precision and Regenerative Medicine and Ionian Area - (DiMePRe-J), University of Bari Aldo Moro, AUOC Policlinico Di Bari, University Hospital Policlinico Di Bari, Piazza Giulio Cesare, 11, 70124 Bari, Italy; 2 Unit of Arterial Hypertension “Anna Maria Pirrelli”, Department of Precision and Regenerative Medicine and Ionian Area - (DiMePRe-J), University of Bari Aldo Moro, AUOC Policlinico Di Bari, University Hospital Policlinico Di Bari, Piazza Giulio Cesare, 11, 70124 Bari, Italy; 3 Department of Advanced Biomedical Science, Hypertension Research Center, Federico II University of Naples, Via Pansini 5, 80131 Naples, Italy; 4 UOC Di Medicina Interna Ospedaliera, AUOC Policlinico Riuniti Di Foggia, Viale Pinto, 1, 71122 Foggia, Italy; 5 UOC Di Medicina Generale, Ospedale “C. Poma” Di Mantova, ASST Mantova, Str. Lago Paiolo, 10, 46100 Mantua, Italy; 6 Unit of Internal Medicine, Terni University Hospital, Viale Tristano Di Joannuccio, 05100 Terni, Italy

**Keywords:** COVID-19, Arterial hypertension, Arterial stiffness, Hypertension-mediated organ damage

## Abstract

COVID-19 infection induces widespread endotheliitis, with long-term effects on the vascular system and arterial hypertension that are still under investigation. This study verified the possible correlation between COVID-19 infection and blood pressure control. In the multicenter study presented here, we enrolled 185 hypertensive patients with a positive nasopharyngeal swab for SARS-CoV-2 in the 6 months prior to the follow-up visit (Group 1, 90F, age 63.38 ± 10.29 years) and 97 hypertensive patients consistently negative for SARS-CoV-2 (Group 2, 49F, age 64.20 ± 10.79 years), overlapping in terms of age, sex, weight, and Body Mass Index (BMI). We evaluated the follow-up visit within 12 months (T1) and the one prior to the infection (T0). Group 2 had a higher percentage of patients with dyslipidemia and a significantly lower percentage of patients with hypertensive cardiomyopathy (HTN-CM) compared to Group 1. In Group 1, we observed a significant decrease in the number of smokers and an increase in the diagnosis of HTN-CM at T1, with a significant increase in systolic values (134.30 ± 14.93 mmHg vs 132.40 ± 16.19 mmHg, *p* < 0.05) and a decrease in diastolic values (78.30 ± 8.77 mmHg vs 79.63 ± 8.41 mmHg, *p* < 0.05) compared to T0. Moreover, there was a significant increase in the number of antihypertensive drugs taken at T1 in Group 1, particularly angiotensin-receptor blockers, compared to T0. Finally, at T1 in Group 1, we observed a significant increase in pulse wave velocity (PWV) compared to T0. These findings were absent in Group 2. Patients who contracted the COVID-19 infection showed increased vascular stiffness, hence higher blood pressure values, and needed a greater number of medications for blood pressure control.

**Supplementary Information:**

The online version contains supplementary material available at 10.1007/s11739-025-04138-4.

## Introduction

Hypertension along with diabetes is the most common comorbidity in patients with COVID-19 infection [1, 2].

The presence of these cardiovascular risk factors markedly increases the risk of the COVID‐19 disease progression to its most severe forms. Indeed, the pre‐existing structural and functional alterations of the organs affected by long‐lasting high blood pressure as well as metabolic alterations weaken their resistance to the COVID-19 virus. In addition, the most common complication of these risk factors is the development of atherosclerosis, an inflammatory process that may adversely interact with the virus [3].

Since the beginning of the pandemic COVID-19, it has quickly become evident that despite the SARS-CoV-2 infection affects primarily the respiratory system, the virus exerts its effects on multiple organs, i.e., heart, lungs, kidneys, liver, and lymph nodes, with sizeable consequences for the vascular system as a whole [4].

The SARS-CoV-2 virus uses angiotensin-converting enzyme 2 (ACE2), a key element of the renin–angiotensin–aldosterone system (RAAS), to facilitate its entry into cells [5]. ACE2 is expressed on the endothelial cells of both arterial and venous vessels primarily of the lung, heart, and kidney. Angiotensin II, the main effector of RAAS, acts on the cardiovascular system by binding to AT1 receptors, through which it activates vasoconstriction, inflammation, and fibrosis pathways. Under physiological conditions, ACE2 gives a counter-regulatory role in RAAS by converting angiotensin II to angiotensin 1–7; it also functions on angiotensin I by converting this to angiotensin 1–9 [6]. Both converting peptides have powerful anti-inflammatory, antioxidant, and antifibrotic properties. The SARS-CoV-2 virus restrains the expression of ACE2 on the cell surface, leading to a reduction of the protective role of the enzyme to the endothelium and other organs [4–7]. Also, patients with COVID-19 show markedly elevated level of angiotensin II [4, 8]. Overall, this viral infection results in the activation of the RAAS with its endothelium-damaging function profile. [9] At the beginning of the pandemic, the use of RAAS inhibitors was a topic of debate, as both ACE inhibitors and angiotensin II receptor blockers (ARBs) result in an over-expression of ACE2 on the cell surface that can (in theory) increase susceptibility to infection. Numerous studies have shown that RAAS inhibitors improve endothelial dysfunction [8, 10].

It is therefore plausible the existence of a bidirectional relationship between COVID-19 and endothelial cell dysfunction. On the one hand, the endothelium is, in fact, a target of SARS-CoV-2 virus that induces intracellular damage, including mitochondrial dysfunction, massive cytokine production, microthrombosis, and immune system abnormalities, peaking in a pro-thrombotic state and systemic vascular damage [11, 12]. On the other hand, the damaged and dysfunctional endothelium promotes the activation of the coagulation cascade and abnormal and dysregulated immuno-inflammatory response, promoting the evolution of the infection toward its most clinically severe forms [13]. Importantly, emerging evidence suggests that COVID-19-related endotheliopathy, hypercoagulability, and low-grade inflammation may persist for months after the acute infection, sustaining a pro-thrombotic milieu and increasing long-term cardiovascular risk [14].

Large artery stiffening may in principle be one of the COVID‐19 infection damages with a greater chance to persist after disease recovery. Arterial stiffness is a vascular aging phenomenon that refers to a loss of arterial compliance or changes in artery wall characteristics. According to The European Society of Hypertension (ESH), carotid–femoral pulse wave velocity (PWV) is the gold standard for measuring large-artery stiffness [15, 16].

Clinical studies demonstrated that SARS-CoV-2 is associated with an increased risk of a persistent elevation in blood pressure requiring new or intensified antihypertensive treatment during hospitalization [17–20].

We focused on possible effects of the COVID-19 on arterial stiffness and blood pressure control in hypertensive patients.

## Methods

We conducted a multicenter, observational cohort study involving 282 hypertensive patients enrolled in five Italian hospitals (Bari University Hospital, Naples University Hospital, Foggia University Hospital, Mantova Hospital, and Terni University Hospital) from 2021 to 2023. The inclusion criteria were: *i)* documented history of arterial hypertension, *ii)* follow-up visit after the initial SARS-CoV-2 test, and *iii)* availability of detailed clinical records, including prior and current antihypertensive therapy.

The primary endpoint of the study was the change in blood pressure between baseline (T0) and follow-up (T1).

The secondary end-points included: change in carotid–femoral pulse wave velocity, variation in the prevalence of hypertensive cardiomyopathy, modification in the number of antihypertensive medications required, changes in ankle–brachial index (ABI), and correlations among vascular parameters.

### Study population

Participants were divided into two groups based on their SARS-CoV-2 infection status:

- Group 1 (*n*. = 185): patients with a positive nasopharyngeal swab for SARS-CoV-2 within at least 6 months prior to the follow-up visit.

- Group 2 (*n*. = 97): patients with a consistently negative nasopharyngeal swab for SARS-CoV-2 during the same period (controls).

Both groups were matched for age, sex, weight, and body mass index (BMI) to ensure comparability of baseline characteristics.

Among Group 1, eight patients required hospitalization, including two who were admitted to the intensive care unit (ICU). In addition, 5 patients received domiciliary oxygen therapy. In the remaining cases, only nasopharyngeal swab positivity was documented, as detailed information on mild or moderate symptoms was not systematically collected. We acknowledge that asymptomatic or untested infections cannot be completely excluded in the control group.

### Data collection

Clinical and demographic data were collected at the two time points:

- T0 (pre-infection): The visit prior to SARS-CoV-2 infection, within 12 months before the follow-up (retrospective review of clinical records).

- T1 (follow-up): The visit conducted at least 6 months after the confirmed SARS-CoV-2 infection for Group 1 or an equivalent follow-up period for Group 2.

Data collected included detailed medical history, comorbidities, cardiovascular risk profile, and current antihypertensive therapy. We also recorded the presence of hypertensive cardiomyopathy (HTN-CM) as indicated in the international guidelines at the time of ethical committee approval[16], defined according to echocardiographic criteria, including presence of left-ventricular hypertrophy (LVH) and/or evidence of grade II or more of diastolic dysfunction.

### Blood pressure measurement

Blood pressure was measured at each visit using a standardized protocol. Measurements were taken in a seated position after 5 min of rest, using a calibrated automated sphygmomanometer. Three consecutive readings were recorded, and the mean of the second and third measurements was calculated for both systolic and diastolic blood pressure [16].

### Assessment of arterial stiffness

This was assessed by measuring PWV, an established indicator of vascular stiffness. PWV was measured using a validated noninvasive device (PulsePen) which calculates the velocity of the pressure wave between the carotid and femoral arteries [21]. The measurements were performed by trained personnel, and the mean of two consecutive measurements was recorded. An increase in PWV is considered a marker of increased arterial stiffness that correlates with cardiovascular risk in hypertensive patients [21].

### Ankle–brachial index measurement

ABI was assessed non-invasively using the ABI SYSTEM 100PWV device (Boso – Bosch & Sohn GmbH, Jungingen, Germany) [22]. Measurements were performed in accordance with the current guidelines, with patients in the supine position after a resting period, and ABI was calculated as the ratio of systolic blood pressure at the ankle to that at the brachial artery [23].

### Echocardiographic evaluation

Transthoracic echocardiography (TTE) was performed for assessment of HTN-CM at both T0 and T1. TTEs were carried out using commercially available phased-array machines following a standardized protocol. LVH was identified by prognostically validated sex-specific cut-off values for LV mass/height (LVMi): > 47 g/m2.7 in women and > 50 g/m2.7 in men, respectively [24, 25]. LV end-diastolic dimension was ratiometrically normalized for height [20]. Relative wall thickness was calculated as the ratio between posterior wall thickness and LV internal radius at end-diastole and considered increased if ≥ 0.43 [16, 25]. LV systolic function was assessed by LV ejection fraction [5, 26], and diastolic dysfunction by current guidelines [16, 25, 27].

### Treatments analysis

Changes in antihypertensive therapy were evaluated by recording the number and class of medications at T0 and T1. The classes included:

- Angiotensin-converting enzyme inhibitors (ACEIs)
- Angiotensin II receptor blockers (ARBs)
- Beta-blockers
- Calcium channel blockers
- Diuretics.

### Ethical approval

The study was conducted in accordance with the ethical standards laid down in the 1964 Declaration of Helsinki and its later amendments. The protocol was reviewed and approved by the Independent Ethics Committee of the Azienda Ospedaliero-Universitaria Consorziale Policlinico (Approval No. 0089041/26/10/2021). Written informed consent was obtained from all participants prior to inclusion in the study.

### Sample size calculation

The primary endpoint for sample size estimation was the between‑group difference in the change of blood pressure. Assuming a clinically meaningful difference of 5 mmHg between COVID‑19 and control groups [28], a common standard deviation of 8 mmHg [25], a two‑sided *α* = 0.05, and 95% power, the required sample size per group was 67 participants/group.

Therefore, 134 participants in total would be sufficient under equal allocation. Our actual sample (185 vs 97; total *n* = 282) exceeds this threshold, thus ensuring sufficient power for the predefined endpoint.

### Statistical analyses

These were conducted using IBM SPSS Statistics, version 25.0 (IBM Corp., Armonk, NY, USA). A two-tailed *p* value < 0.05 was considered statistically significant.

A comprehensive descriptive analysis was initially performed to characterize the study population. The distribution of continuous variables was assessed using the Kolmogorov–Smirnov test.

To compare baseline characteristics between patients with a history of SARS-CoV-2 infection and those without, we employed univariate analyses using the appropriate statistical tests based on variable type and distribution. Specifically, the independent samples t test was applied for normally distributed continuous variables, and the Mann–Whitney U test for non-normally distributed variables. Categorical variables were compared using the chi-square test or Fisher’s exact test, as appropriate.

Longitudinal changes within each group were analyzed by comparing baseline (T0) and follow-up (T1) values. For continuous variables, paired samples t tests were used in the case of normally distributed data, while the Wilcoxon signed-rank test was used for non-parametric distributions.

To explore the differential impact of SARS-CoV-2 infection on the trajectory of clinical and instrumental parameters, we computed delta values (Δ = T1–T0) for each continuous variable and compared them between groups using either the independent samples t test or Mann–Whitney U test, depending on the data distribution.

Exploratory stratified analyses were also performed by sex, age categories, and baseline blood pressure control status to identify potential effect modifiers. Within each stratum, comparisons followed the same statistical methodology outlined above.

To better understand if COVID-19 is the key determinant in our endpoint, we perform first a univariate regression between swab positive detection and each end point (difference systolic blood pressure, diastolic blood pressure, number of antihypertensive drugs, pulse wave velocity, and ankle–brachial index between the two time points). To point out if other factors may influence the result, a multivariate linear regression was adjusted in a model that includes sex, age at T0, and difference in eGFR and BMI between the two time points.

## Results

At T1, the mean age of Group 1 was 63.38 ± 10.29 years, with 48.6% females (Table 1), while of Group 2 it was 64.20 ± 10.79 years, with 50.5% females. The prevalence of major cardiovascular risk factors, including diabetes mellitus, dyslipidemia, and smoking status, was also similar between the two groups at baseline, except for a higher incidence of dyslipidemia in Group 2 (*p* < 0.05) (Tables 2 and 3).

**Table 1:** Anthropometric parameters of the patients in study group and controls at T0 and T1 visit

	Group 1 (*n*. = 185)		Group 2 (*n*. = 97)	
	T0	T1	T0	T1
Male/female	95/90		48/49	
Age (years)	62.39 ± 10.21	63.38 ± 10.29	63.70 ± 10.50	64.20 ± 10.79
Weight (cm)	79.37 ± 17.25	78.84 ± 15.61	82.85 ± 17.97	82.98 ± 19.02
Height (cm)	165.10 ± 9.48	165.50 ± 9.57	166.40 ± 11.03	165.40 ± 11.06
Body Mass Index (Kg/m^2^)	28.98 ± 4.98	28.80 ± 4.67	29.41 ± 4.38	29.74 ± 4.90
Abdominal circumference (cm)	102.70 ± 13.75	103.40 ± 13.32	102.90 ± 10.82	104.60 ± 13.42
Body surface area (m^2^)	1.89 ± 0.24	1.90 ± 0.22	1.95 ± 0.26	1.71 ± 0.69
Heart rate (bpm)	67.99 ± 10.41	69.56 ± 9.40	67.46 ± 9.10	70.21 ± 11.53

No significance between the Groups at any timepoint

**Table 2:** History and medication of the patients in Group 1 and Group 2 at T0 and T1 visit

	Group 1 (*n*. = 185)		Group 2 (*n*. = 97)	
	T0	T1	T0	T1
History				
Smokers	102	86*	62^^	62°°°
Dyslipidemia	60	60	76^^^	65°°°
Diabetes	94	85	18	20
Heart disease	116	139*	72^^^	73°
Chronic obstructive pulmonary disease	13	13	1	2
Arrhythmias	13	13	14	18
Medications				
Angiotensin-converting enzyme inhibitors	40	53	20^^^	17°
Angiotensin II receptor blocker	87	106*	65^^^	68°°
Calcium channel blockers	46	62	35	38
Beta-blockers	62	76	37	36
Alpha-blockers	3	15	17	10
Thiazide	54	60	30	37
Chlorthalidone	4	2	0	0
Furosemide	4	6	0	0
Indapamide	3	7	5	5
Median number of antihypertensive drugs	2 [1–3]	2 [1–4]*	2 [1–3]	2 [1–3]
Statins	81	105*	54	65
Ezetimibe	7	16	7	12
Acetylsalicylic acid	36	46	28	26
Antiaggregants	16	21	10	10
Warfarin	3	7	2	5
Oral antidiabetes	60	59	10	12
Insulin	4	6	2	2
Other antidiabetes	26	20	6	6

Group 1 T1 vs T0, *p < 0.05

Group 2 T0 vs Group 1 T0, ^^*p* < 0.005, ^^^ p < 0.001

Group 2 T1 vs Group 1 T1, °*p* < 0.05, °°*p* < 0.005, °°°*p* < 0.001

**Table 3:** Laboratory parameters of the patients in Group 1 and Group 2 at T0 and T1 visit

	Group 1 (*n*. = 185)		Group 2 (*n*. = 97)	
	T0	T1	T0	T1
Creatinine (mg/dL)	0.90 ± 0.21	0.92 ± 0.30	0.88 ± 0.19	0.84 ± 0.19
Estimated Glomerular Filtration Rate (mL/min)	84.57 ± 14.80	84.24 ± 15.97	82.03 ± 17.63	84.97 ± 17.40
Glycemia (mg/dL)	96.14 ± 17.72	98.00 ± 19.76	92.87 ± 19.16	96.30 ± 21.98
Total Cholesterol (mg/dL)	182.00 ± 34.37	180.90 ± 40.97	176.30 ± 38.83	166.30 ± 34.47
High Density Lipoprotein (mg/dL)	52.94 ± 13.71	54.73 ± 13.53	53.49 ± 12.73	53.25 ± 14.71
Low Density Lipoprotein (mg/dL)	106.5 ± 32.33	105.40 ± 37.25	101.60 ± 36.82	96.79 ± 32.81
Triglycerides (mg/dL)	124.50 ± 75.93	114.40 ± 51.69	119.40 ± 59.29	102.90 ± 39.82**#**
Urate (mg/dL)	5.32 ± 2.83	5.18 ± 1.46	5.09 ± 1.21	5.29 ± 1.16
Hemoglobin (g/dL)	14.01 ± 1.40	13.81 ± 1.51	14.52 ± 1.43	14.51 ± 1.21
White Blood Cell (× 10^3/uL)	6.54 ± 1.79	6.98 ± 2.65	6.76 ± 1.83	6.56 ± 1.60
Neutrophils (%)	57.89 ± 9.92	58.48 ± 8.81	60.82 ± 8.82	60.65 ± 8.32
Lymphocytes (%)	30.96 ± 7.94	30.65 ± 7.91	29.48 ± 7.70	28.46 ± 7.52
Platelets(× 10^3/uL)	230.10 ± 58.34	234.50 ± 62.41	233.50 ± 65.20	245.00 ± 60.96

Group 2 T1 vs T0, # *p* < 0.05

### Baseline characteristics and prevalence of hypertensive cardiomyopathy

At T0, a significant difference was observed in the prevalence of HTN-CM between the two groups. Group 1 showed a markedly higher prevalence of HTN-CM compared to Group 2 (*p* < 0.05), suggesting a more advanced stage of hypertensive disease at baseline. This difference in HTN-CM prevalence remained significant even after adjusting for potential confounders, such as age, sex, and duration of hypertension (Table 2).

### Clinical outcomes and hypertensive cardiomyopathy

Group 1 showed a significant increase in the prevalence of newly diagnosed hypertensive cardiomyopathy at T1 (*p* < 0.05), suggesting that COVID-19 infection may accelerate the progression of cardiac structural changes in hypertensive patients. No such increase was seen in Group 2 (Table 4), where the prevalence of HTN-CM remained stable.

**Table 4:** Echocardiographic parameters of the patients in Group 1 and Group 2 at T0 and T1 visit

	Study group (*n*. = 185)		Controls (*n*. = 97)	
	T0	T1	T0	T1
Interventricular septal (mm)	12.00 ± 2.72	12.42 ± 2.74	12.62 ± 1.57	12.97 ± 1.35
Posterior Wall in diastole (mm)	11.82 ± 2.77	12.20 ± 2.95	11.68 ± 1.59	11.91 ± 1.67
Left-Ventricular dimension in diastole (mm)	47.19 ± 4.10	46.22 ± 5.66	46.51 ± 4.31	46.99 ± 4.62
Left-Ventricular end-Diastolic Volume (mL)	95.69 ± 32.87	85.99 ± 39.62	119.40 ± 31.11	103.50 ± 25.80
Left-Ventricular Mass (g/m^2^)	210.30 ± 71.91	214.50 ± 72.68	213.60 ± 65.00	224.70 ± 60.48
Left-Ventricular Mass indexed (g/m^2^)	107.30 ± 32.45	119.30 ± 41.69	111.70 ± 27.26	124.20 ± 29.10
Left-Ventricular Mass indexed h2.7 (g/m^2.7^)	55.07 ± 19.38	60.30 ± 21.50	55.30 ± 12.96	60.10 ± 13.38
Relative Wall Thickness	0.44 ± 0.07	0.56 ± 0.81*	0.52 ± 0.08^^^	0.51 ± 0.07
Ejection Fraction (%)	61.25 ± 5.69	61.60 ± 5.77	62.47 ± 5.12	61.34 ± 4.65
Aortic root (mm)	30.53 ± 3.96	30.92 ± 4.85	30.96 ± 4.89	32.42 ± 4.40
Left Atrium volume (mL)	60.17 ± 21.30	61.37 ± 23.00	64.37 ± 20.35	62.15 ± 21.30
Left Atrium volume/Body Surface Area (mL/m^2^)	28.80 ± 12.64	31.70 ± 11.46	34.79 ± 8.39	30.87 ± 11.10
Velocity E (cm/s)	66.82 ± 19.19	63.57 ± 20.02	63.36 ± 16.97	66.43 ± 15.86
Velocity A (cm/s)	75.76 ± 17.10	73.35 ± 19.74	78.50 ± 19.21	80.76 ± 17.92
Velocity E/Velocity A	0.95 ± 0.63	0.88 ± 0.29	0.85 ± 0.31	0.85 ± 0.25
Velocity e’ (cm/s)	8.14 ± 2.37	8.07 ± 1.81	7.57 ± 1.27	7.58 ± 1.84
Velocity E/Velocity e’	8.13 ± 4.35	8.15 ± 7.15	8.14 ± 2.12	9.38 ± 2.99
Right basal ventricular diameter (mm)	30.28 ± 3.15	31.25 ± 5.19	31.23 ± 4.64	33.65 ± 5.76
Right Ventricular Area (cm^2^)	15.67 ± 2.22	15.71 ± 4.61	21.22 ± 7.35	22.12 ± 4.91
Right Atrium volume (mL)	38.67 ± 16.42	47.50 ± 22.49	55.14 ± 16.29	52.31 ± 14.39
Right Atrium Area (cm^2^)	15.40 ± 6.09	17.30 ± 5.09	18.50 ± 3.16	17.32 ± 3.26
Inferior Vena Cava (mm)	17.74 ± 5.42	15.79 ± 2.99	15.38 ± 3.02	14.26 ± 2.95
Tricuspid Regurgitant Velocity (cm/s)	2.52 ± 0.34	2.38 ± 0.50	2.15 ± 0.56	2.06 ± 0.41
Pulmonary Artery Pressure systolic (mmHg)	31.57 ± 5.14	29.46 ± 9.42	28.87 ± 8.59	23.25 ± 7.67
Tricuspid Annular Plane Systolic Excursion (mm)	23.46 ± 3.35	24.26 ± 4.20	25.59 ± 6.57	26.05 ± 4.00

Group 1 T1 vs T0, * *p* < 0.05

Group 2 T0 vs Group 1 T0, ^^^ *p* < 0.001

### Changes in blood pressure at follow-up

**Fig. 1:**
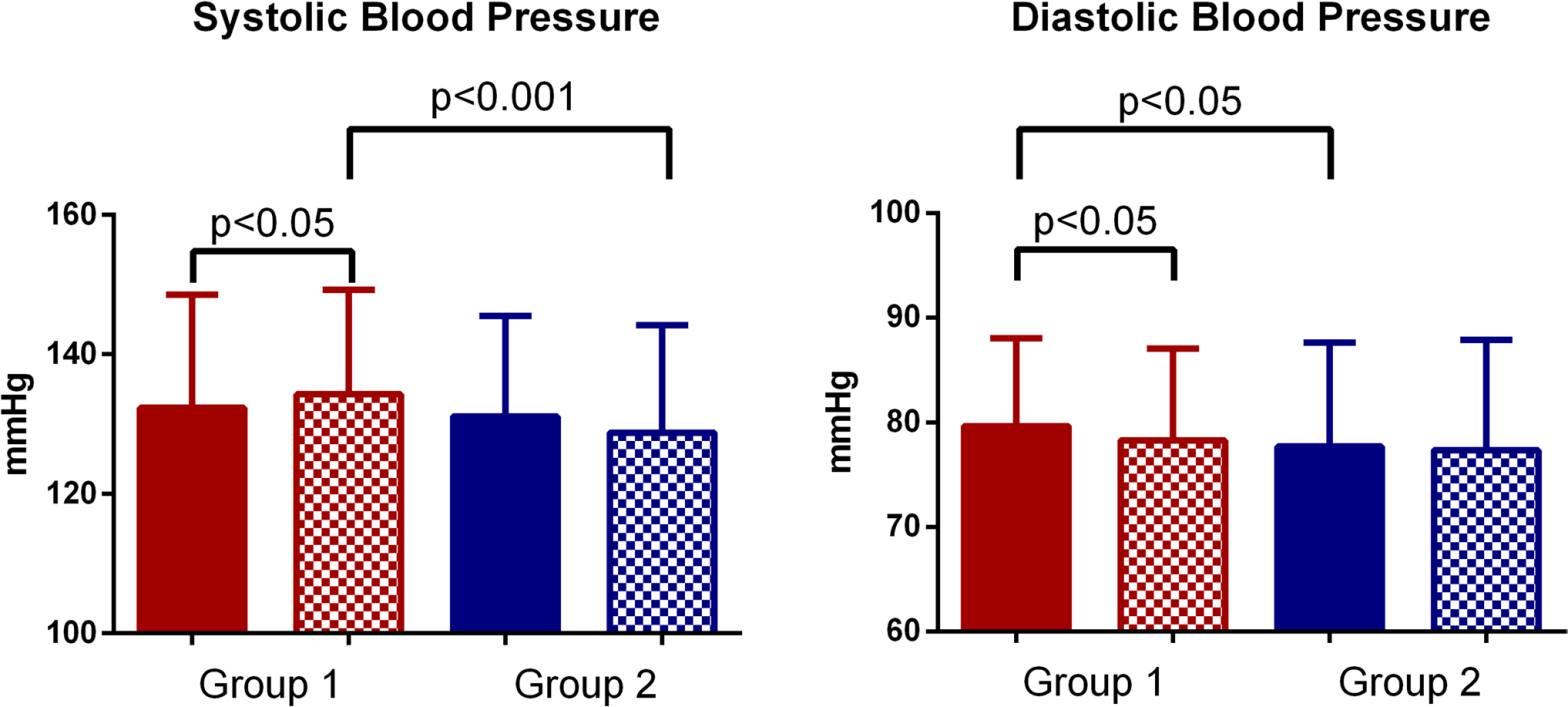
Changes in systolic and diastolic blood pressure before (T0) and after (T1) SARS-CoV-2 infection. Plain bars indicate T0 evaluation, while dotted bars indicated T1. Red bars are for the Group 1, while blue bars are for Group 2

At T1, Group 1 exhibited a significant increase in systolic blood pressure compared to the baseline (134.30 ± 14.93 mmHg vs 132.40 ± 16.19 mmHg, *p* < 0.05), while diastolic blood pressure showed a slight but significant decrease (78.30 ± 8.77 mmHg vs 79.63 ± 8.41 mmHg, *p* < 0.05) (Fig. 1). The rise in systolic blood pressure suggests a trend toward worsening blood pressure control in hypertensive patients with a history of SARS-CoV-2 infection. In contrast, Group 2 did not show significant changes in either systolic or diastolic blood pressure between T0 and T1.

### Impact on vascular stiffness

**Fig. 2:**
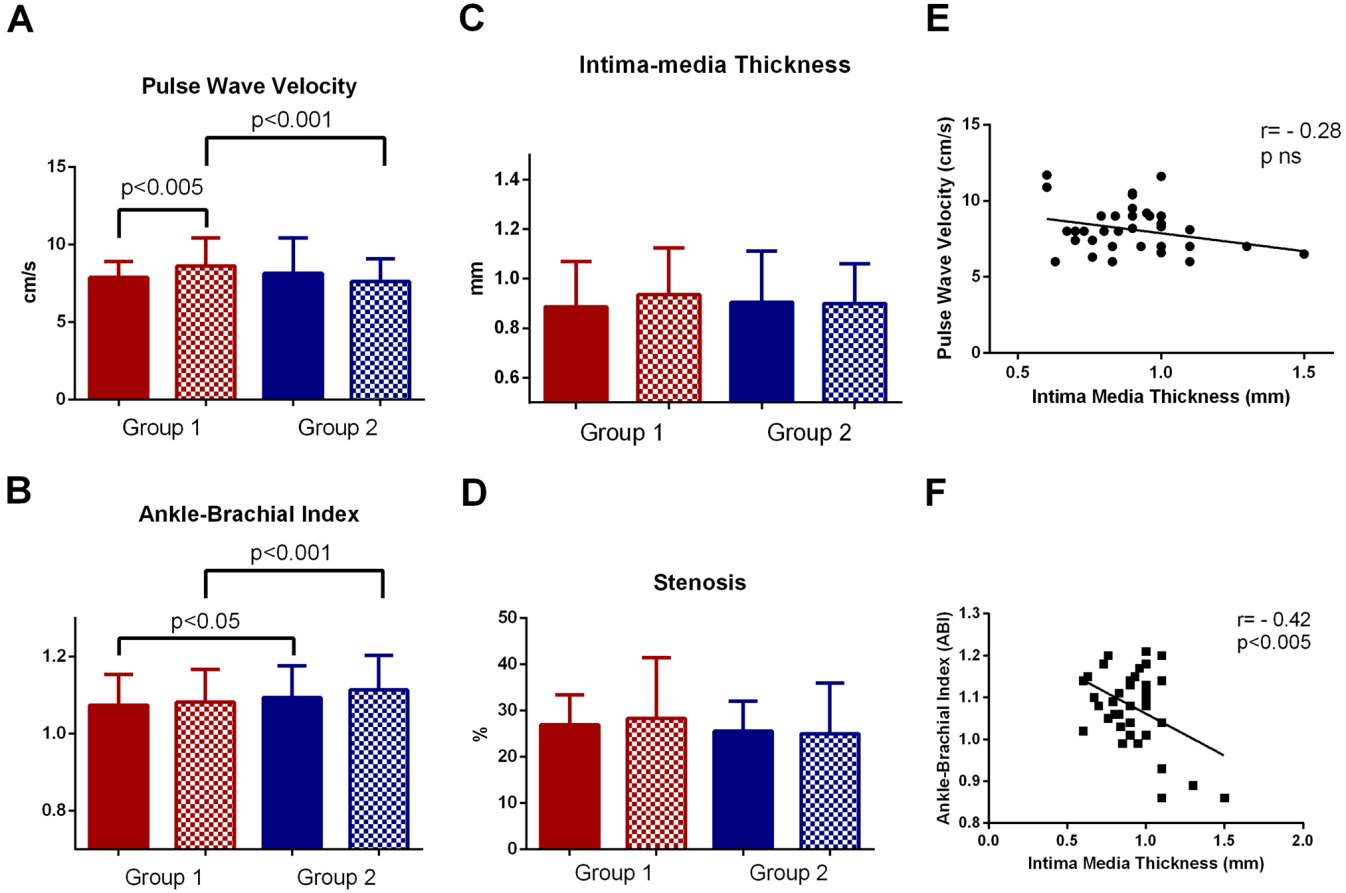
Markers of vascular damage before (T0) and after (T1) SARS-CoV-2 infection. **A** Carotid–femoral pulse wave velocity (PWV). **B** Ankle–brachial index (ABI). **C** Carotid intima–media thickness (IMT). **D** Carotid stenosis (%) measured using the European Carotid Surgery Trial (ECST) method. **E–F** Correlation between IMT and PWV (**E**), and between IMT and ABI (**F**), at T1 in the COVID-19 group. While IMT did not correlate with PWV, it showed a significant inverse correlation with ABI. Plain bars represent baseline (T0) values and dotted bars represent follow-up (T1). Red bars correspond to COVID-19 patients (Group 1), and blue bars correspond to controls (Group 2)

At T1, PWV was significantly increased in Group 1 compared to T0 (8.62 ± 1.83 m/s vs 7.87 ± 1.13 m/s, *p* < 0.05), indicating a progressive deterioration of arterial elasticity post-infection. By contrast, Group 2 did not show significant changes in PWV (Fig. 2). Similar results were seen for ABI (Fig. 2B), while Intima-Media Thickness (IMT) or stenosis (%) did not change (Fig. 2C and D). Finally, there was a significant correlation between IMT and ABI but not PWV (Fig. 2E and F) at T1 in the Group 1.

### Antihypertensive drugs and changes in medication

One notable finding was the significant increase in the number of antihypertensive medications prescribed to Group 1 patients at follow-up. At T1, the median number of antihypertensive drugs per patient was increased (*p* < 0.05). In particular, the use of ARBs showed a substantial rise (*p* < 0.01).

In contrast, Group 2 did not show significant changes in the overall number of antihypertensive medications between T0 and T1. The stable medication regimen aligns with the lack of significant changes in blood pressure and PWV, reinforcing the hypothesis that the alterations observed in Group 1 are directly related to the effects of SARS-CoV-2 infection.

### Determinants in end-points

Univariate regression results in a significant inverse correlation between the difference in systolic blood pressure and the Group, supporting the results that indicate an increase in SBP in Group 1. No other significant linear regression was found (Beta − 5.57 [confidence interval − 10.28 to − 0.86], *p* = 0.02). However, to evaluate possible effects of confounding factors or interferences, multivariate regression was performed. Also in this analysis persists a statistically significant inverse correlation between the Group and the change in SBP. Due to this result, we further performed the same multivariate regression to the other end-points determinants. Interestingly, in multivariate regression, there was a significant correlation to in DBP and ABI (directly) and PWV (inversely), while no correlation was found in change of number of antihypertensive drugs (Supplementary Table).

## Discussion

**Fig. 3:**
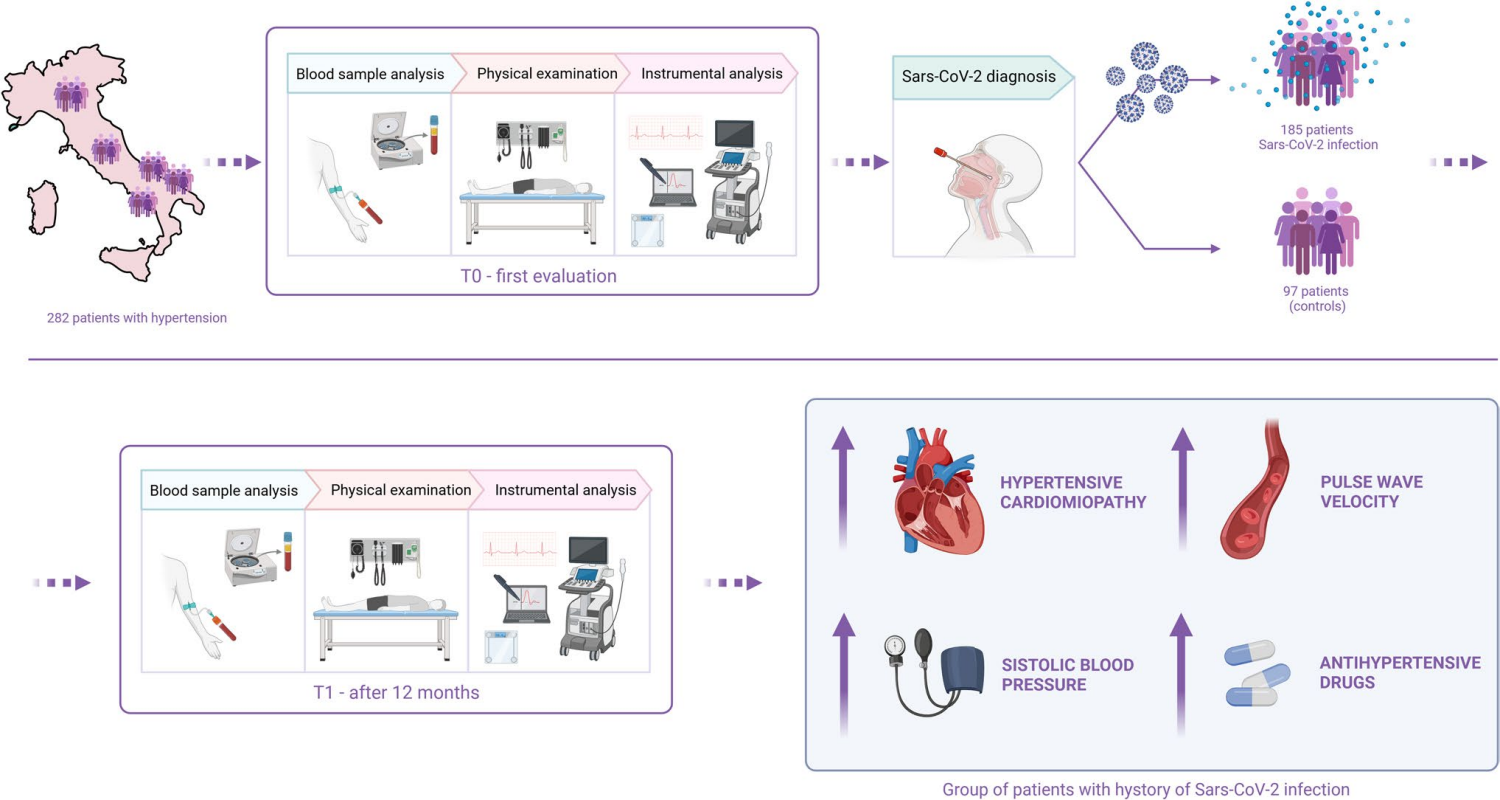
Graphical summary of the multicenter study design and key findings. Created in BioRender. Cicco, S. (2025) https://BioRender.com/bzv7wmr

Here, we show that hypertensive patients with a history of COVID-19 infection have a significant increase in systolic blood pressure and arterial stiffness, and a need for an increased number of antihypertensive medications compared to hypertensive patients without prior infection. The increase in vascular stiffness, as evidenced by higher PWV values, suggests that COVID-19 may induce persistent endothelial dysfunction, which could contribute to long-term cardiovascular complications in these patients (Fig. 3).

It is unlikely that SARS-CoV-2 infection, occurring only a few months prior, directly induced de novo hypertensive cardiomyopathy. A more plausible explanation is that infection-related endothelial dysfunction, vascular inflammation, and increased arterial stiffness acted as accelerators of pre-existing subclinical cardiac damage in hypertensive patients, thereby favoring an earlier manifestation of left-ventricular hypertrophy. This interpretation is supported by prior evidence of COVID-19-induced endotheliitis [29] and by the established association between arterial stiffness, hypertensive target organ damage, and cardiovascular outcomes [16].

A recent retrospective cohort examined the long-term effects of the COVID-19 infection on blood pressure among 5355 nonhospitalized patients who were followed up 12.5 ± 0.4 months after recovery. Both systolic and diastolic blood pressures were significantly higher post-infection. There were 456 (17%) patients with new-onset hypertension and 456 (14%) with exacerbated hypertension requiring an intensified antihypertensive regimen [30].

In our study, a reduction in diastolic blood pressure emerged. Tentatively, this might be explained by the increase in large-artery stiffness, which is the most important pathophysiological determinant of increase of systolic blood pressure and reduction of diastolic blood pressure [31].

Although the between-group difference in mean systolic blood pressure was small (134 vs 132 mmHg), even minor but sustained increases can have clinical impact in hypertensive patients. Epidemiological evidence has shown that the relationship between systolic blood pressure and cardiovascular risk is continuous, without a clear threshold [32, 33]. By extrapolation, a persistent difference of few mmHg may still translate into a measurable increase in long-term cardiovascular risk, particularly in an already high-risk population. In our cohort, this modest increase was accompanied by a significant intensification of antihypertensive therapy, reinforcing its clinical significance.

Meta-analysis by Zuin et al. showed that the COVID-19 recovered subjects presented an increased risk of new-onset hypertension compared to subjects who did not experience the infection. As demonstrated by the meta-regression, this risk was directly influenced by age, female sex, and cancer, and resulted higher in the early post-acute phase of the infection [34].

Earlier studies have shown, in different patient groups, a significantly higher arterial stiffness and PWV in COVID-19 patients compared to control groups: Schnaubelt et al*.* showed differences in arterial stiffness between acutely ill patients with and without COVID-19 [35]; Peng et al. [36] shown effects of COVID-19 infection on vascular function in young college students who did not require hospitalization. Their small pre-post-controlled study’s findings indicate that throughout the course of the COVID-19 infection, there was a significant increase in carotid–femoral pulse wave velocity (cfPWV) with a mean difference of 0.44 m/s (*p* = 0.022) and systolic blood pressure with a mean difference of 3.24 mmHg (*p* = 0.047). Diastolic blood pressure also exhibited an increase, though it was not statistically significant, with a mean difference of 2.65 mmHg (*p* = 0.068)[36].

An increasing number of clinical studies suggest that apart from the acute phase of the disease, the SARS-CoV-2 virus may cause prolonged and extended effects on blood vessels. Lambadiari et al.[37] conducted a case–control prospective study including 70 patients 4 months after the COVID-19 infection, matched with 70 newly diagnosed and untreated patients with hypertension of similar age and sex (positive control) and 70 healthy individuals. Compared to controls, both COVID-19 and hypertensives had higher PWV (carotid–femoral PWV 12.09 ± 2.50 vs. 11.92 ± 2.94, *p* = 0.7 vs. 10.04 ± 1.80 m/s, *p* = 0.036) and impaired left- and right-ventricular function (GLS). These results support that the SARS-CoV-2 virus causes endothelial and vascular dysfunction that persists 4 months after the onset of infection and is, thus, linked to reduced cardiac performance.

Interestingly, in Group 1, we observed a significant correlation between IMT and ABI but not with PWV at follow-up. This result, although exploratory, highlights the concept that different vascular indices capture distinct pathophysiological pathways: IMT and ABI are primarily markers of atherosclerotic burden and peripheral vascular disease, while PWV reflects central arterial stiffness and large-artery function [38]. The selective association of IMT with ABI may therefore suggest that COVID-19-related vascular damage is more closely linked to structural and peripheral alterations rather than uniformly involving central arterial stiffness.

Moreover, the correlation between COVID-19 infection and our endpoint, especially the multivariate results, strongly supports our study hypothesis. In particular, there was an association between COVID-19 infection and blood pressure worsening and vascular stiffness. Our results demonstrate in a similar period but using a different method that COVID-19 induce a vascular damage like Bruno et al. [39] recently published.

There are several proposed mechanisms regarding the development of cardiovascular outcomes after acute COVID-19 illness; persistence of viral reservoirs might lead to chronic inflammation, and resultant chemokines might cause damage via reactive oxidative species. Endothelial dysfunction causes loss of barrier function and antithrombotic properties. The potential of the RAAS and kinin–kallikrein system (KKS) pathways to induce a cytokine or bradykinin storm has been proposed as a mechanism for the development of cardiovascular consequences of long COVID. [30].

Taking all these data together with our results we can suggest having a particular caution to patients who were hypertensives and experienced infection of SARS-CoV-2. The increase in arterial stiffness may induce a higher incidence of major cardiovascular events, even in patients that were previously in good blood pressure control [40]. A possible evaluation of COVID-19-induced change in inflammatory cytokines and oxidative stress related to the increase in vascular stiffness may help to understand the molecular mechanism of the described results. This evaluation may also help in identifying possible target for personalized therapy or evaluating the possible role of SGLT2-inhibitors or GLP1-RA as drugs able to protect vascular health.

This study has several limitations. First, this is an observational study; therefore, it is possible to establish a correlation between the worsening in blood pressure control and SARS-CoV-2 infection but not a specific causality. However, it is intelligible that it could be difficult to have a different nature of similar study. Second, we performed a 1-year follow-up. Longer evaluation is needed to establish long-term cardiovascular change. Third, despite self-reported drug assumptions was recorded, no urine evaluation for metabolites test was performed. Furthermore, a limitation of our study is the lack of detailed data on antiviral therapy during the acute phase of COVID-19, including possible drug–drug interactions such as the temporary discontinuation of statins during Paxlovid administration. Finally, another possible limitation is the potential misclassification of controls, since asymptomatic or unrecognized infections may have occurred during the study period. Nevertheless, the use of documented swab positivity as a diagnostic standard reflects the methodology adopted in most clinical studies during the pandemic.

## Conclusions

This study provides evidence that hypertensive patients who have contracted COVID-19 have more difficult blood pressure control overtime compared to those without a history of infection. The increased vascular stiffness, higher systolic blood pressure, and greater need for antihypertensive medications suggest that the SARS-CoV-2 virus may have a lasting impact on the vascular system. These findings underscore the need for long-term cardiovascular monitoring in patients who have contracted the COVID-19 disease, particularly those with pre-existing hypertension.

## Supplementary Information

Below is the link to the electronic supplementary material.

Supplementary file1 (DOCX 9 KB)

## Data Availability

The data are not publicly available due to ethical restrictions.
