# Supplementary material for: COVID-19 induces greater difficulty in blood pressure control due to increased arterial stiffness
Source: Intern Emerg Med. 2025 Nov 5;20(8):2421–32. doi: 10.1007/s11739-025-04138-4 (PMC12672848; doi:10.1007/s11739-025-04138-4)
Supplement: Supplementary file 1 — Supplementary file1 (DOCX 9 KB) [file 11739_2025_4138_MOESM1_ESM.docx]

Supplementary Table: multivariate regression to covid infection history and end-point variables.

| Value | Beta [confidence interval] | p |
| --- | --- | --- |
| Systolic Blood Pressure | -4.68 [-9.26 : -0.11] | 0.045 |
| Diastolic Blood Pressure | 3.37 [0.15 : 6.72] | 0.049 |
| Pulse Wave Velocity | -0.99 [-1.63 : -0.35] | 0.003 |
| Ankle-Brachial Index | 0.03 [0.002 : 0.06] | 0.037 |
| Number of anti-hypertensive drugs | -0.10 [-0.30 : 0.11] | 0.332 |

Multivariate regression model to covid infection history includes sex and age at T0 and change between T1 and T0 in body mass index and estimated glomerular filtration rate.
